# Development of Functional Thyroid C Cell-like Cells from Human Pluripotent Cells in 2D and in 3D Scaffolds

**DOI:** 10.3390/cells10112897

**Published:** 2021-10-26

**Authors:** Kwaku Dad Abu-Bonsrah, Donald F. Newgreen, Mirella Dottori

**Affiliations:** 1 The Murdoch Children’s Research Institute, Royal Children’s Hospital, Parkville, VIC 3052, Australia; 2 Department of Paediatrics, University of Melbourne, Parkville, VIC 3010, Australia; 3 Department of Biomedical Engineering, Department of Anatomy and Neurosciences, University of Melbourne, Parkville, VIC 3010, Australia; 4 Illawarra Health and Medical Research Institute, School of Medicine, Molecular Horizons, University of Wollongong, Wollongong, NSW 2522, Australia

**Keywords:** pluripotent stem cells, endoderm, disease modelling, thyroid C cells

## Abstract

Medullary thyroid carcinoma contributes to about 3–4% of thyroid cancers and affects C cells rather than follicular cells. Thyroid C cell differentiation from human pluripotent stem cells has not been reported. We report the stepwise differentiation of human embryonic stem cells into thyroid C cell-like cells through definitive endoderm and anterior foregut endoderm and ultimobranchial body-like intermediates in monolayer and 3D Matrigel culture conditions. The protocol involved sequential treatment with interferon/transferrin/selenium/pyruvate, foetal bovine serum, and activin A, then IGF-1 (Insulin-like growth factor 1), on the basis of embryonic thyroid developmental sequence. As well as expressing C cell lineage relative to follicular-lineage markers by qPCR (quantitative polymerase chain reaction) and immunolabelling, these cells by ELISA (enzyme-linked immunoassay) exhibited functional properties in vitro of calcitonin storage and release of calcitonin on calcium challenge. This method will contribute to developmental studies of the human thyroid gland and facilitate in vitro modelling of medullary thyroid carcinoma and provide a valuable platform for drug screening.

## 1. Introduction

Thyroid cancer is a malignant tumour of the endocrine system and the most common of the endocrine cancers, accounting for 1% of newly diagnosed cancers [1]. Malignant thyroid cancers can be categorised as differentiated of follicular cell origin (papillary, follicular, and Hṻrthle cell), less differentiated of C cell origin (medullary thyroid carcinoma or MTC) and mostly hereditary [2], and undifferentiated and aggressive (anaplastic) [3].

Statistics in both the USA and Australia show that the incidence of thyroid cancer detection is increasing faster than that of other solid tumours with a 3:1 preponderance of females (Australian Government Cancer Statistics) [4]. In the USA, about 40,000 female and 13,000 male new cases were estimated regarding individuals diagnosed with thyroid cancer in 2018, with about 1.2% of adults having this diagnosis at some time in their lifetime [5,6]. Reports show the rise in incidence of differentiated thyroid cancers in children and teens as well as adults [7,8]. Thyroid cancers may show comorbidities; for example, pheochromocytoma and parathyroid adenoma frequently occur with MTC in multiple endocrine neoplasia type 2 [9], and the incidence of MTC is elevated in patients with the neural defect of the distal colon, Hirschsprung disease [10]. 

MTC results mainly from somatic or germline mutation in the RET (rearranged during transfection) proto-oncogene [11] and contributes to about 3–4% of thyroid cancers [12]. It is a distinct thyroid cancer which originates from thyroid C cells and has a strongly metastatic potential. C cells are located outside the thyroid follicles and are hence referred to as parafollicular. The C cells produce calcitionin, a hormone that regulates Ca^2+^ metabolism by lowering blood Ca^2+^ levels [13,14], in opposition to the effects of parathyroid hormone (Poole and Reeve, 2005). 

Treatment of MTC is typically thyroidectomy and often also with local lymph node removal. This radical surgery dates back over 100 years and is intricate with real danger of collateral damage [15,16]. Radiotherapy is frequently recommended as follow-up treatment. Tyrosine kinase inhibitors vandetanib and cabozantinib, which inhibit the RET growth factor receptor, have been approved by the U.S. Food and Drug Administration; however, RET was not the intended target, and these drugs also have powerful inhibitory effects on other receptors such as VEGFR (vascular endothelial growth factor receptor), EGFR (epidermal growth factor receptor), MET (mesenchymal to epithelial transition), KIT (KIT proto-oncogene, receptor tyrosine kinase), FLT3 (FMS-like tyrosine kinase 3), TIE-2 (tyrosine kinase with immunoglobulin-like loops and epidermal growth factor homology domain-2), TRKB (tropomyosin receptor kinase B), and AXL (AXL receptor tyrosine kinase) [17,18]. Because of these side effects, they are used with caution and only for the most aggressive forms of MTC, and therefore improvements in pharmacological therapeutics would be welcomed.

Disease modelling in the human cell context provides the platform to better understand and find appropriate therapeutic measures [19,20,21,22]. Generating a disease model specifically for MTCs requires a differentiation protocol for the affected cell type, thyroid C cells.

**Figure 1:**
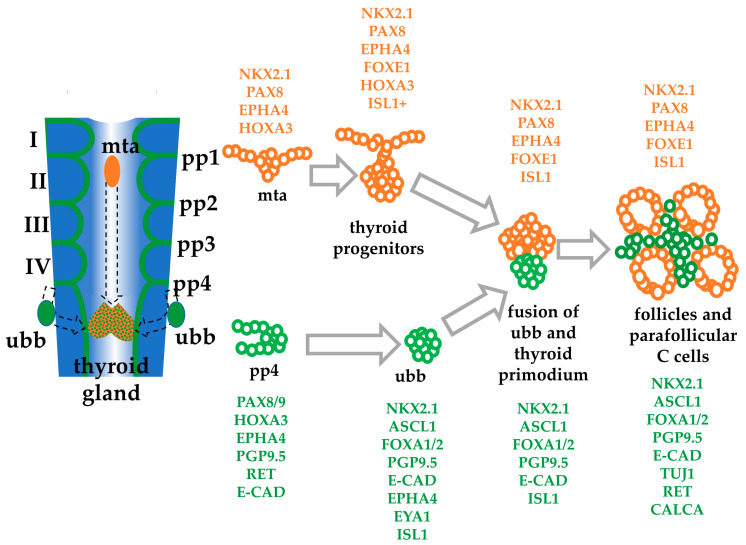
Development of the thyroid organ. Scheme of the development of the pharynx showing the origin and morphogenetic movements of the medial thyroid *anlage* (mta) from the midventral pharynx, and the ultimobranhial body (ubb) from pharyngeal pouch 4 (pp4). These migrate and fuse to form the follicular and parafollicular C cells, respectively, of the mammalian thyroid gland. These moieties remain separate in other vertebrates. Roman numbers indicate the pharyngeal arches. Key genes are indicated in orange (thyroid follicular cell differentiation) and green (thyroid parafollicular C cell differentiation) [25,38].

There has been much research on the differentiation of derivatives of the foregut endoderm, including lung progenitors [23] and thymus progenitors [24]. Work on the thyroid focusses on follicular cells, and methods are available for production and differentiation of these cells in vitro from pluripotent cells [25,26]. There is less information about the differentiation of C cells, and even their embryonic origin has had to be drastically revised. Unlike follicular cells, thyroid C cells do not arise from the thyroid *anlage* but rather from the ultimobranchial body (ubb) during embryonic development [27] and differentiate into functional C cells in mammals once the ubb fuses with the thyroid [28,29,30] (Figure 1). In non-mammalian vertebrates, this fusion does not occur [31]. Previously, C cell progenitors were viewed as being of neural crest origin [32], and this fitted with the permanent or transient expression of a suite of markers associated with neurons such as serotonin (5HT) receptor, somatostatin receptor, calcitonin gene-related peptide (CGRP), β-III tubulin (TUJ1), PGP9.5, and many others, as well as neurotransmitter storage vesicles [29,33]. However, recent research on the embryonic origin, development, and differentiation of mouse and human thyroid C cells in vivo proved that in mammals C cells originate from foregut endoderm [29,34] and can be classed as enteroendocrine cells.

The use of 3D cell culture methods has opened many avenues to better understand how cells reorganise to form tissues, how different cell types interact, and how cells respond to external stimuli such as drugs and hormones. These techniques have been used in stem cell biology to study organogenesis and in cancer biology to study angiogenesis, metastasis, and drug screening since 3D models mimic the in vivo environment in terms of attaining appropriate tissue architecture with improved cell differentiation and functionality [35,36,37].

Here, we show a novel differentiation method for deriving functional human thyroid C cell-like cells in vitro via foregut endoderm-like intermediate steps followed by neural-like induction conditions using 2D and 3D scaffold techniques. These cells should prove useful models for detailed studies of human C cell differentiation as well as providing human cellular models for diseases such as MTC which affect this cell type.

## 2. Materials and Methods

### 2.1. Ethics Statement

All experiments were performed with the approval from the Murdoch Children’s Research Institute Institutional Biosafety Committee 226-2015 PC2. Human Stem Cell studies were performed with approval from University of Melbourne Human Ethics, ID 1545384, 0605017 and 1545394.

### 2.2. Human Embryonic Stem Cells, hESC Culture, and Thyroid C Cell Differentiation in 2D Cultures

**Figure 2:**
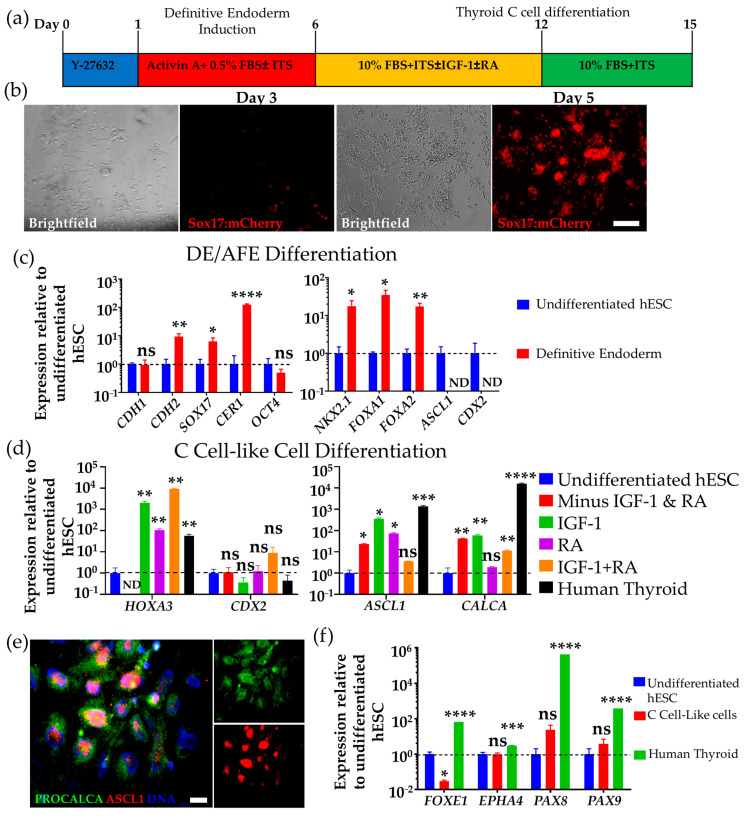
HESC-derived cells express definitive endoderm/anterior foregut endoderm (DE/AFE) and thyroid C cell markers. (**a**). Differentiation protocol of hESCs to DE/AFE-like cells and further to thyroid C cell-like cells. (**b**) Microscopy analysis of DE/AFE-like cells induced after 3 and 5 days exposure to activin A showing the expression of *SOX17* detected by mCherry expression using the H9 hESC-SOX17 mCherry reporter line. Scale bar: 200 µm. (**c**) qPCR analysis of DE/AFE-like cells at day 6 (red bars) normalized to undifferentiated hESC (blue bars), showing downregulation of pluripotency marker *OCT4*, no expression of posterior foregut marker *CDX2*, upregulation of diverse early DE/AFE markers, and no expression of the later C cell markerASCL1. (**d**) qPCR analysis of DE/AFE-like cells differentiated to thyroid C cell-like cells at day 15 normalized to undifferentiated hESC (blue bar) comparing different conditions (no IGF1 and no RA, red bar; IGF1 only, green bar; RA only, purple bar; IGF1 and RA, orange bar) with human thyroid (black bar) (commercial cDNA library). Consistent with thyroid C cell lineage is elevated expression of *ASCL1*, *HOXA3*, and *CALCA* and depression of *CDX2* (posterior foregut lineage). (**e**) Immunofluorescence of H9 hESC-derived DE/AFE-like cells differentiated with IGF-1 (day 15 of differentiation), showing co-expression of C cell lineage markers, *ASCL1* (red) and *PROCALCITONIN* (green). Scale bar: 50 µm. (**f**) qPCR analysis of differentiated thyroid C cell-like cells differentiated with IGF-1 for 6 days (day 15 of differentiation; red bar) normalized to undifferentiated H9 hESC (blue bar) comparing the expression of thyroid follicular and C cell transcription factors. Human thyroid was used as a positive control (green bar). FOXE1 is confined to the follicular lineage while *PAX8, PAX9*, and *EPHA4* are expressed in both lineages but only early and transiently in the C cell lineage. N.D., not detected; n.s., not significant, * *p* < 0.05, ** *p* < 0.01, *** *p* < 0.001, **** *p* < 0.0001. *n* = 3 independent experiments (H9 hESC). Error bars represent mean ± SEM. Insulin-like Growth Factor 1 (IFG1); Retinoic acid (RA); definitive endoderm (DE); anterior foregut endoderm (AFE); human embryonic stem cells (hESC).

hESCs (H9, WiCell; HES3, ESI International; 007 iPSC, kindly provided by Alice Pébay, Centre for Eye Research Australia) were cultured to 80–90% confluence and harvested using EDTA-PBS buffer for 4–6 min. The buffer was gently removed, and 2 mL of medium was gently added to harvest the cells into a 15 mL Falcon tube, and 10 µL was counted using a haemocytometer (Thermo Fisher Scientific, North Ryde, Australia). Cells were seeded as a monolayer at 1 × 10^5^ on Matrigel-coated organoid culture dishes with TESR™-E8™ complete media supplemented with 3.2 µg/mL (10 µM) Y-27632 (Sigma-Aldrich, Castle Hill, Australia) and cultured at 37 °C and 5% CO_2_ in a humidified incubator overnight; this was recorded as day 0 (see Figure 2a). On day 1, the media was changed to DMEM/F12 supplemented with 0.5% foetal bovine serum (FBS) and 100 ng/mL (3.82 nM dimer) HumanKine Activin A (Miltenyi Biotec, Macquarie Park, Australia) with 1X ITS-A (insulin–transferrin–selenium–sodium pyruvate, Thermo Fisher Scientific) and cultured at 37 °C and 5% CO_2_ in a humidified incubator for 5 days. Medium was replaced on days 3, 4 and 5. On day 6, the media was changed to DMEM/F12 supplemented with 10% FBS, 1% pen–strep, 1X ITS-A with 2 ng/mL (261.5 pM) recombinant human IGF-1 protein, carrier-free (R&D systems - In Vitro Technologies, Lane Cove West, Australia) or 3 ng/mL (10 nM) retinoic acid, RA (Sigma-Aldrich) for 6 days (until day 12) or with both IGF-1 and RA. Medium change was done every other day until day 12, where the cells were cultured without IGF-1 or RA for 3 days, until day 15. The cells spontaneously formed domes/nodules around days 10–11. On day 15, cells were assayed for calcitonin by ELISA (see below) before cells were harvested for RNA extraction. Parallel cultures for immunofluorescence staining were fixed and stained following the protocols outlined below.

### 2.3. Thyroid C Cell Differentiation Using 3D Gel Matrigel Scaffold Cultures

hESCs were harvested as discussed above, and 1 × 10^5^ cells were resuspended in 35% Matrigel (*v*/*v*) (Corning-In Vitro Technologies, Lane Cove West, Australia) in DMEM/F12 supplemented with 0.5% FBS and 1X ITS-A, and seeded into 2 wells of a 96 well plate (50,000 cells per well); then they were placed in the incubator 37 °C for 60 min for the gel to cast before we topped the well up with DMEM/F12 supplemented with 0.5% FBS, 1X ITS-A, and 100 ng/mL activin A and cultured them at 37 °C and 5% CO_2_ in a humidified incubator. This was counted as day 1, and culture proceeded for 5 days. Medium change was done after 2 days and then every day. On day 6, medium was changed to DMEM/F12 supplemented with 10% FBS, 1X ITS-A, and 2 ng/mL IGF-1 for 6 days. Medium change was done every day until day 12, where the cells were cultured without IGF-1 for 3 days. On day 15, cells were stimulated with Ca^2+^ for calcitonin secretion before harvesting cells for RNA extraction.

### 2.4. Immunocytochemistry/Immunofluorescence

Cells, both 2D and 3D, were fixed with 4% paraformaldehyde (PFA) for 15 min at room temperature and washed three times with PBS. The cells were then permeabilised for 30 min with 0.2% Triton-X and 3% horse serum in PBS with 0.2% azide and further blocked with 1% horse serum in PBS-azide for 30 min. The cells were then washed once with PBS-azide and stained with primary antibodies per the manufacturer’s instructions and incubated at 4 °C overnight. Antibodies are listed in Table S1. Cells were then washed three times with PBS with a 5 min interval between PBS changes. For detection, cells were incubated with appropriate fluorochrome-conjugated secondary antibodies for 2–3 h at room temperature (see Table S1). Residual secondary antibodies were removed, and the cells were washed twice with PBS before counterstaining the nuclei with 10 ng/mL 4′6-diamidino-2-phenylindole dihydrochloride, DAPI (Sigma-Aldrich). Cells were then mounted in antifade mounting medium (90% glycerol (pH 8) with 200 mM DABCO; Sigma-Aldrich) and viewed using a Zeiss LSM 780 (Carl-Zeiss-Strasse, Oberkochen, Germany) confocal microscope. 

For confocal optical sections, 3D differentiated cells in Matrigel were fixed in 4% PFA for 1 h at room temperature and washed three times with PBS. Immunostaining was done as above.

For cryostat sections, 3D differentiated cells in Matrigel were fixed with 4% PFA for 1 h at room temperature and placed in 30% sucrose in CMF-PBS overnight. Cells were then embedded in Tissue Tek OCT Medium in Tissue Tek cryomoulds (ProSciTech, Thuringowa, Australia) and frozen in dry ice-cooled isopentane. Sections were cut using a Leica CM 1900 cryostat microtome and collected on Superfrost microscope slides (Biolab Scientific, Auckland, New Zealand) coated with poly-L-lysine. Slides were stained as above. See the Supplementary Materials for antibody lists.

False antibody binding was checked by using a first antibody not reactive with human antigens and by omitting first antibodies. Reactivity of other antibodies were checked by using a known target, human kidney organoids supplied by Professor Melissa Little and Dr. Jessica Vanslambrouck.

### 2.5. RNA Extraction and qPCR

Total RNA was extracted from cultured cells using TRIzol (Thermo Fisher Scientific) and purified lysate by acid-phenol chloroform and later recovered by isopropanol/ethanol precipitation. Before cDNA synthesis, extracted RNA was digested with DNaseI (Promega, Sydney, Australia) following the manufacturer’s instructions to remove residual DNA and synthesized using the Bioline SensiFAST™ cDNA Synthesis Kit (BIO-65054, Bioline (Aust) Pty Ltd- Eveleigh, Australia). We used the Human Thyroid Total RNA (Takara Bio-sourced from Scientifix Pty Ltd, Clayton, Australia) as a positive control. Briefly, 30–100 ng total RNA was converted into cDNA following the manufacturer’s directions. Reactions were performed using cDNA converted from 30–100 ng of RNA, 50 nM of each primer, and AccuPower^®^ 2X GreenStar Master Mix Solution (Bioneer Pacific, Kew East, Australia) in a total volume of 10 μL. Primers for qPCR analysis are listed in Table S3. For Taqman Assay analysis, reactions were performed using cDNA converted from 30–100 ng of RNA, 1X of Taqman Probe, and GoTaq^®^ Probe qPCR Master Mix Solution (Promega) in a total volume of 10 μL. All runs were in triplicate and more than 3 (*n* ≥ 3) independent experiments. *GAPDH* housekeeping gene was used for data normalization when the relevant gene was undetectable in the control population. Cultured hESCs were used as a calibration standard and relative gene expression changes were calculated using the 2^−δδt^ method. See the Supplementary Materials for primer lists.

### 2.6. FACs Sorting and Analysis

To detect intracellular proteins, we fixed cells with 2% PFA for 10 min and permeabilised them with 0.1% Triton-X in 1% horse serum, blocked them with 1% horse serum, and incubated them with primary antibodies in blocking buffer at 4 °C overnight. After they were washed with CMF-PBS and centrifuged, the appropriate secondary antibodies were added for 1 h and the cells were washed with CMF-PBS, pelleted, and resuspended in CMF-PBS containing 2% FBS. The cells were then strained (40 μm mesh; BD Falcon, North Ryde, Australia) and events were acquired with BD FACs X-20 Fortessa (BD Biosciences, Frenchs Forest, Australia). Data was analysed using CellQuest (BD Biosciences) and FCS Express 4 Flow (Denovo Software, Glendale, CA, USA).

### 2.7. Calcitonin Production and Secretion

To determine whether hESC-derived thyroid C cells produce calcitonin, we homogenized cells generated from hESCs in 2D culture in 200 µL sonication buffer consisting of 50 mM Tris-HCl pH 7.5, 0.1% SDS, and 2% EDTA (Thermo Fisher Scientific). Qsonica sonicator (Qsonica, Newtown, CT, USA) was used to sonicate the cells on ice with 1 burst at 25% amplitude for 5–10 s. Sonicated lysate was centrifuged for 5 min at 10,000 rpm, and the supernatant was used to analyse calcitonin concentration by ELISA. 

To determine whether hESC-derived thyroid C-like cells are capable of secreting calcitonin on Ca^2+^ challenge, we washed cells generated from hESCs in either 2D or 3D conditions three times with a Krebs–Ringer buffer containing 10 mM HEPES. Cells were incubated with a 100 mM CaCl_2_ solution in magnesium-free PBS at 37 °C for 90 min. Supernatants were then collected and analysed for calcitonin as above, and the remaining cells were then collected for RNA extraction and qPCR analysis. 

### 2.8. ELISA Assay

Calcitonin levels were measured using a Human Procalcitonin ELISA kit (Thermo Fisher Scientific) following the manufacturer’s instructions. Briefly, all reagents, standards, and samples were brought to room temperature (18–25 °C) before use. Then, 100 µL of each procalcitonin standard, and sonicated and centrifuged sample was added to the supplied wells (duplicates per sample), which were pre-coated with procalcitonin antibody. Wells were then covered and incubated for 2–3 h at room temperature with gentle shaking. The solution was discarded, and wells were washed 4 times with 1X Wash Buffer. After the last wash, the remaining Wash Buffer was removed by aspiration and blotting by inverting against clean paper towels. Then, 100 µL of 1X prepared biotinylated antibody was added to each well, which were incubated for 1 h at room temperature with gentle shaking. The solution was discarded, and the wells were washed 4 times with 1X Wash Buffer. Then, 100 µL of prepared streptavidin-HRP solution was added and incubated for 45 min at room temperature with gentle shaking. The solution was discarded, and wells were washed 4 times with 1X Wash Buffer followed by adding 100 µL of TMB substrate to each well. The plates were incubated for 30 min at room temperature in the dark with gentle shaking, and the reaction was stopped by adding 50 µL of the stop solution to each well. Absorbance was measured on an ELISA plate reader set at 450 nm. The calculation of results was done using the ELISA software analysis tool online (elisaanalysis.com accessed on 2 April and 7 May 2017). The amounts of calcitonin were normalized by measuring total intracellular protein using a BCA protein assay kit (PIERCE, Thermo Fisher Scientific) as described below.

### 2.9. BCA Protein Assay (PIERCE)

Briefly, 25 µL of each standard or the sample were added in replicate into a microplate well (working range = 20–2000 µg/mL) (Thermo Fisher Scientific). Then, 200 µL of the sample was added to each well and mixed thoroughly on a plate shaker for 30 s. The plate was covered and incubated at 37 °C for 30 min. Plates were then cooled to RT before measuring the absorbance at 562 nm on a plate reader. We then subtracted the average 562 nm absorbance measurement of the blank standard replicates from the 562 nm measurements of all other individual standard and unknown sample replicates and prepared a standard curve by plotting the average blank-corrected 562 nm measurement for each BSA standard vs. its concentration in µg/mL. The standard curve was used to determine the protein concentration of each unknown sample.

### 2.10. Statistical Analyses

Data were analysed by using one-way ANOVA (when analysing only one variable-gene expression) and unpaired *t*-test with Welch’s correction. Values were expressed as mean ± SEM. Changes were deemed significant if the *p*-value was <0.05. Statistical significance is indicated as follows: * *p* < 0.05, ** *p* < 0.01, and *** *p* < 0.001. Graphs were drawn using GraphPad Prism v7.

## 3. Results

### 3.1. Differentiation of hESCs to Definitive Endoderm and Anterior Foregut Endoderm-like Cells

Recent research has thrown light on the embryonic origin, development, and differentiation of mouse thyroid C cells in vivo, proving that C cells originate from foregut endodermal cells via the ubb [34]. We modified a previous published protocol on differentiation of definitive endoderm (DE) [39,40] by adding ITS-A to the media to support the growth of the cells in the presence of low FBS [26] (Figure 2a). Three different human pluripotent cell lines, H9 and HES3 hESCs, and 007 iPSCs, were used in our studies.

To confirm the efficiency of our DE differentiation from hESCs, we used the Sox17-mCherry H9 hESC reporter line, provided by Professor Andrew Elefanty and Professor Ed Stanley, and analysed the number of mCherry expressing cells after 3 and 5 days exposure to activin A. The fidelity of mCherry and SOX17 expression has been previously demonstrated [41]. We recorded about 30–40% of cells expressing mCherry after 3 days differentiation with almost all cells (≥90%) expressing mCherry after 5 days differentiation with this protocol (Figure 2b). 

The induced cells were analysed at day 6 for the expression of vertebrate DE markers [42,43] *FOXA2, CER1, CDH2* (N-cadherin), and *SOX17* by qPCR, and we observed an upregulation of these genes together with a downregulation of the pluripotency marker, *OCT4,* while *CDH1* (E-cadherin) was unchanged (Figure 2c). A robust increase in a gene expressed in the anterior foregut endoderm (AFE), *NKX2.1* (also known as thyroid transcription factor 1, *TTF1*) [26], and *FOXA1* [34], was also found. *CDX2* is a marker for posterior foregut identity, and through qPCR analysis, we found that *CDX2* mRNA expression was undetected in the cultures (Figure 2c and Figure S1a,b), consistent with an anterior foregut identity. A similar finding was reported by Wells’ group [44], consistent with long exposure to activin A promoting anterior foregut over posterior identity. The expression of the proneuronal gene *ASCL1* in the hESC-derived DE could not be detected (Figure 2d). This suggests the progenitors are at an early stage of DE differentiation since *ASCL1* expression in mouse C cell development begins later at around E11.5 [45], equivalent to the fifth week (Carnegie stage 14) in human embryos.

### 3.2. Differentiation of Human DE/AFE-like Cells to Thyroid C Cell-like Cells

Next, we sought to differentiate these cells into thyroid C cell-like cells by modifying previously published protocols on lung, hepatocyte, and thyroid progenitor cell differentiation, which involves using FGF2 and BMP4, as well as Wnt and Sonic hedgehog (Shh) factors [26,46,47]. Although the ubb fuses with the thyroid primordium later during mammalian development (see Figure 1), we sought to disfavour the differentiation of thyroid and lung progenitors by increasing the time of activin A exposure and by omitting dexamethasone, BMP4, FGF2, and Wnt agonists which promote thyroid and lung differentiation [26,48]. FGF2 and BMP4 exposure of endodermal lineage cells also promotes the differentiation of hepatocytes and pancreatic cells [49,50], therefore further supporting the omission of both BMP4 and FGF2. Both Shh agonists and antagonists were also omitted from the differentiation method since ectopic expression of Shh favours intestinal lineage differentiation [51]. Furthermore, although the ubb and hence thyroid C cells fail to fuse with the thyroid primodium in Shh-/- mice, their development and differentiation are not affected [52], suggesting Shh is not required. 

Given the extraordinary similarity in transcriptional read-out between neural crest-derived neurons and enteroendocrine cells [45], we used factors such as IGF-1 that have been used in neuronal differentiation of stem cells [53,54], with the idea being that this might favour C cell-like neuroendocrine differentiation of cells already embarked on the DE/AFE pathway. We also used IGF-1 for the differentiation on the basis that IGF-1 activates the PI3K/Akt1 and Ras/MAPK signalling pathways [55], which have been associated with anterior foregut endoderm specification and epithelialisation [56]. Cell interaction with extracellular matrix (ECM) such as laminin and collagen IV interact with PI3K/Akt1 and Ras/MAPK signalling which play a role in the specification of foregut precursors, and inhibition of the PI3K/Akt1 pathway disrupts anterior DE specification [56,57], and therefore we used ECM substrates. In addition, retinoic acid (RA) was tested as it favours many neural differentiations [58], as well as being important in specification of the third and fourth pharyngeal pouches, the origin of the ubb [59].

We differentiated the DE cells in high FBS while supplementing the medium with IGF-1 and retinoic acid (RA) for 6 days and analysed them after 3 days (day 15 total differentiation) on Matrigel-coated surfaces [31,47] (Figure 2a). On the basis of a previously published protocol where primary embryonic C cells were cultured in 5% FBS and 1% chick embryo extract, we employed FBS only, but increased its concentration from 5% to 10% [60]. 

We assessed which condition will lead to an increased expression of ASCL1 and CALCA. The pro-neural gene ASCL1 plays an important but late role in the development and differentiation of C cells, and ASCL1 mutant mice lack C cells, although the thyroid gland develops normally (Figure 2c) [45]. CALCA encodes not only calcitonin, which is specific for C cells, but also calcitonin gene-related peptide (CGRP) and katacalcin by alternative RNA splicing and cleavage of precursor proteins. The development and migration of the ubb also depend on the expression of the paralogs of HOX3—an example is HOXA3 [45,61]. We further analysed which condition will disfavour posterior foregut identity by analysing the expression of *CDX2* mRNA. 

Culture with no addition of growth factors and with the addition of RA only had no effect on mRNA expression of the posterior foregut marker, *CDX2*. Cultured cells treated with IGF-1 led to a reduced expression of *CDX2* (Figure 2c,d) to a similar level to that recorded in the commercial human adult thyroid sample. IGF-1 treatment also led to an increase in *ASCL1* and *CALCA* as compared to the other conditions and a comparable level of *HOXA3* expression comparing it to both IGF-1- and RA-treated conditions. Combining IGF-1 and RA resulted in the overexpression of *CDX2* mRNA as analysed by qPCR (Figure 2d), suggesting a more posterior endodermal patterning. We then confirmed the expression of ASCL1, procalcitonin, E-cadherin, and CGRP by immunostaining (Figure 2e and Figure S1c,d). We also immunostained with Islet1, which has been shown to be a C cell precursor marker (see Figure S1e) [62]. These results are in congruence with previously published findings of the role of IGF-1-activated pathways in thyroid C cell development [57].

Next, in addition to ASCL1 and CALCA, we asked whether the differentiated cells after 6 days of IGF-1 exposure express FOXE1, EPHA4, PAX8, and PAX9, which are highly expressed in the human thyroid in spatiotemporally specific patterns (see Figure 1). The expression of FOXE1 (also known as TTF2, thyroid transcription factor 2) [63] is regulated by the expression of NKX2.1 and PAX8, and its expression downstream of NKX2.1 and PAX8 distinguishes thyroid follicular progenitors from non-follicular cells during development [51,64]. Thyroid progenitors are specified by the expression of PAX8 with EPHA4 during thyroid folliculogenesis [51]. They are expressed ubiquitously at E9.5 in the mouse pharyngeal endoderm [61] but the ubb begins to lose EPHA4 expression after delamination from the pharyngeal pouch followed by complete loss of EPHA4 expression when the ubb merges with the median thyroid *anlage* [65]. Expression of PAX9 also plays an important role in the patterning of the pharyngeal pouches, as well as regulating calcitonin gene transcription [66,67]. To this end, we performed qPCR analyses on these genes which are expressed in human adult thyroid from a commercial cDNA library (see Table S2). The qPCR results show a comparable expression of markers between our differentiated cells and the human thyroid. Although there were higher fold differences in follicular markers FOXE1, PAX8, and PAX9 expression in the human thyroid resource (which was derived from both follicular and parafollicular cells), there was no significant change in *EPHA4* expression in hESC-derived C cell-like cells relative to undifferentiated hESC (Figure 2f). The reduction in *FOXE1* mRNA expression is consistent with the development of C cells and also indicates that the differentiated cells are not follicular thyroid cells [51].

**Figure 3:**
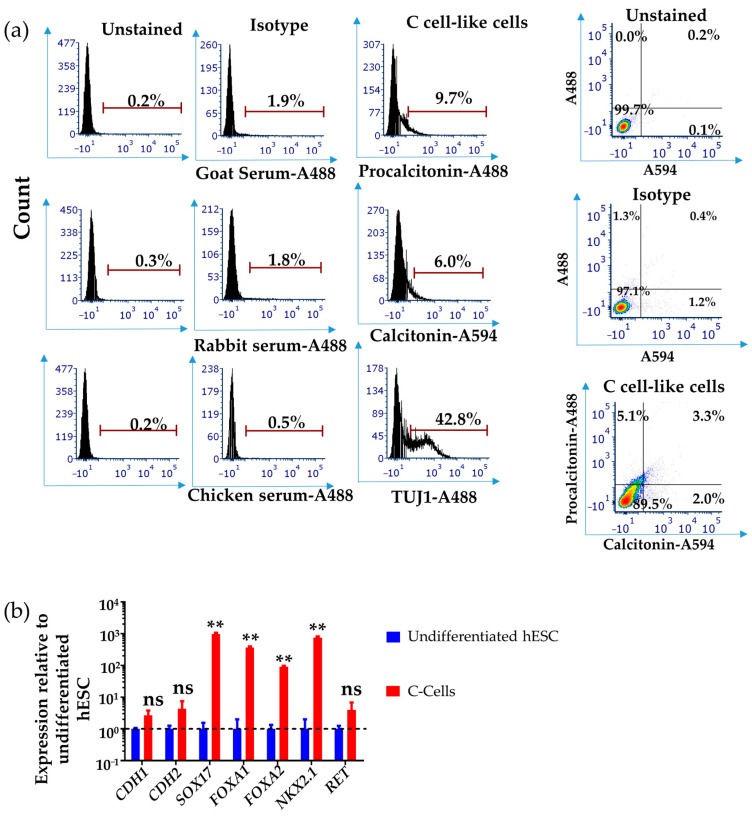
hESC-derived cells express thyroid C cell markers. (**a**) Representative FACS plots of H9 hESC-derived DE/AFE cells differentiated to thyroid C cell-like cells after 3 days of exposure to IGF-1 as analysed using PROCALCITONIN, CALCITONIN, and TUJ1 antibodies. The plot suggests emergence of PROCAL+/CALC+ sub-populations after 3 days of IGF-1 exposure while more than 40% of the cells express TUJ1, a neuronal marker expressed in C cells. (**b**) qPCR analysis of day 15 differentiated thyroid C cell-like cells (red bars) comparing the expression of C cell markers, normalised to undifferentiated hESC (H9, blue bars). Error bars represent mean ± SEM. n.s., not significant, ** *p* < 0.01. *n* = 3 independent experiments. Human embryonic stem cells (hESC).

**Figure 4:**
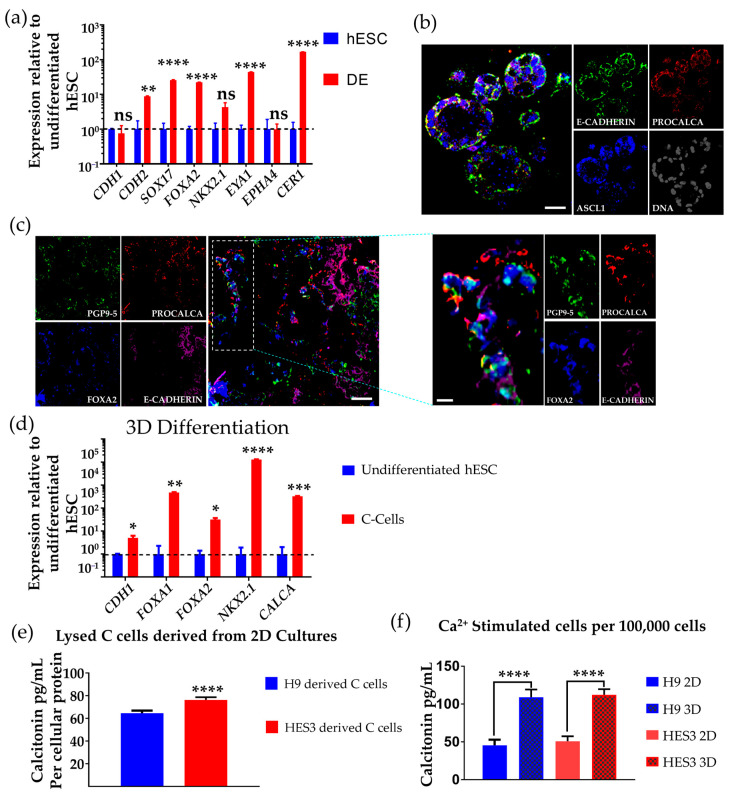
Differentiated human thyroid C cell-like cells produce calcitonin in culture. (**a**) qPCR analysis of 3D differentiation of DE/AFE-like cells (red bars) at day 6 normalized to undifferentiated hESC (blue bars), showing upregulation of DE/AFE markers. *n* = 3 independent experiments (H9 hESC). (**b**) Immunostained confocal optical section of day 15 H9 hESC differentiated thyroid C cell-like cells in 3D Matrigel culture co-expressing *E-CADHERIN* (green), *PROCALCITONIN* (red), and *ASCL1* (blue). Nuclei are shown in grey. Scale bar: 50 µm. (**c**) Immunofluorescence of cryosectioned H9 hESC differentiated thyroid C cell-like cells (day 15) in 3D Matrigel co-expressing *E-CADHERIN* (purple), *PROCALCITONIN* (red), *FOXA2* (blue), and *PGP9.5* (green). Scale bar: 100 µm. (**d**) qPCR analysis of 3D differentiation of C cell-like cells with IGF-1 (day 15 of differentiation) (red bars) normalised to undifferentiated hESC (H9, blue bars). *n* = 3 independent experiments. (**e**) ELISA analysis of differentiated thyroid C cell-like cells (H9 hESC-derived, blue bars; HES3 hESC-derived, red bars) at day 15 of 2D culture showing the production of calcitonin (cells lysed in 200 µL buffer) normalised to the total cellular protein as measured by BCA representing of 0.5–1 × 10^6^ cells. Error bars represent mean ± SEM. *n* = 3 independent experiments. (**f**) Comparison of the secretion of calcitonin by cells differentiated in the 2D (unpatterned bars) and 3D (patterned bars) format (day 15 of differentiation) into the medium (200 µL) as stimulated by 100 mM Ca^2+^ per 100,000 cells (H9 hESC-derived, blue bars; HES3 hESC-derived red bars). Error bars represent mean ± SEM. n.s., not significant, * *p* < 0.05, ** *p* < 0.01, *** *p* < 0.001, **** *p* < 0.0001. *n* = 3 independent experiments. Human embryonic stem cells (hESC); definitive endoderm (DE); anterior foregut endoderm (AFE).

To investigate the efficiency of our differentiation protocol, we performed FACS analyses on the cells after 3 days of IGF-1 differentiation to examine expression of calcitonin, procalcitonin, and TUJ1. The results show that by day 3, approximately 9% of the cells expressed the procalcitonin marker, 6% expressed calcitonin, and more than 40% expressed TUJ1. C cells have been shown to express the neuronal marker TUJ1 during early development at E14.5 in mouse [45,60,61]; therefore, taken together, the results support hESC differentiation towards a thyroid C cell-like lineage by this stage (Figure 3a). Analysis of day 15 differentiated cells by qPCR showed the expression of thyroid C cell markers *FOXA1*, *FOXA2, NKX2.1*, *RET,* and *CALCA* (Figure 3b, and refer to Figure 2d for *CALCA* expression). However, the expression of *SOX17* indicates the continued presence of undifferentiated endodermal progenitors (Figure 3b). We then immunostained for E-cadherin, procalcitonin, and CGRP; these are markers expressed by C cells with procalcitonin being restricted to the C cell lineage [14,31,45]. We show that more than 50% of the cells co-expressed E-cadherin and procalcitonin indicating that this differentiation protocol is generating thyroid C cell-like cells in vitro (Figure 4a,b). We performed qPCR analysis to further support that our differentiation protocol disfavours the differentiation of lung epithelial cells and the results showed the downregulation of lung epithelial cell specific genes, *SFTPC* and *CC10* (see Figure S2c). We further immunostained for thyroid follicular marker PAX8 to investigate the efficiency of the differentiation protocol, and the negative result indicated the absence of thyroid follicular cells. Staining on the positive control (kidney organoids) indicates the validity of this antibody (see Figure S3a,b).

### 3.3. Differentiation of hESCs to Neuroendocrine Thyroid C Cell-like Cells in 3D Matrigel

The interaction in vitro between ECM and cells regulates cell differentiation by providing a microenvironment mimicking that found in vivo, and this has been shown in vitro in neural differentiation [68], kidney differentiation [69], small intestine differentiation [70], colon differentiation [71], development of pancreatic beta islet cells [72], and development of thyroid follicular cells [73]. We hypothesised that differentiating hESCs to thyroid C cell-like cells in 3D format in ECM (as compared to 2D on ECM) will support more efficient generation of thyroid C cell-like cells and the production of calcitonin.

Dissociated hESCs were embedded in Matrigel and differentiation following the same protocol as for monolayer culture (see Figure 2a). At day 6, the cells were analysed by qPCR for the DE/AFE markers and showed expression of *SOX17, FOXA2, NKX2.1*, *CER1*, *EYA1*, and *CDH2* (Figure 2c). We then further differentiated the cells to C cell-like cells using the same IGF-1 protocol as with the monolayer cultures. We performed confocal optical section and cryosection analysis of 3D cultures for E-cadherin, procalcitonin, and ASCL1 and the neuroendocrine marker PGP9.5, and found co-expression at day 15 in more than 70% of the cells [31,61] (Figure 4b,c). It is noteworthy here that C cells can form follicle-like structures, and this has been shown to be its appearance of the ubb during development in some mammalian and non-mammalian species [28,74]. Immunostaining of 3D cultures also showed expression of E-cadherin, procalcitonin, PGP9.5, and FOXA2. Of particular importance for C cell differentiation is procalcitonin, as expression of this is restricted to later stages in the thyroid C cell lineage [14,31,34,45]. qPCR analysis of differentiated cells with IGF-1 in 2D culture (day 15 of differentiation) showed the expression of thyroid C cell markers *FOXA1*, *FOXA2*, *NKX2.1*, *RET*, and *CALCA* (Figure 3b, and refer to Figure 2d for *CALCA* expression). qPCR analysis of differentiated cells at equivalent time and in 3D culture showed the expression of C cell markers *FOXA1*, *FOXA2*, *NKX2.1*, and *CALCA* (Figure 4d). 

### 3.4. Determination of Calcitonin Production and Secretion on Ca^2+^ Challenge

The distinctive function of thyroid C cells is the production of calcitonin, a hormone that regulates Ca^2+^ metabolism by lowering blood Ca^2+^ levels. To assess the potential functionality of the hESC-derived C cell-like cells, we first had to confirm whether these cells were producing calcitonin, and to that end, cells were lysed in 0.2 mL of lysis buffer after 15 days differentiation in 2D conditions and analysed by ELISA. The ELISA results were then normalised to the total cellular protein analysed by BCA assay. The results showed that the cells were producing 12.8 to 20.2 pg (64–101 pg/mL in a volume of 200 µL culture medium) of calcitonin per 0.5–1 × 10^6^ cells with the cells derived from HES3 cell line at 76.3 pg/mL (15.3 pg of calcitonin per 0.5–1 × 10^6^ cells) and the H9 hESC cell line at 64.5 pg/mL (12.9 pg of calcitonin per 0.5–1 × 10^6^ cells) (Figure 4e). 

We then investigated whether the cells were capable of secreting calcitonin into the medium when challenged with Ca^2+^ by adding calcium chloride (final concentration of 100 mM) to the cells. Assessment of the supernatant by ELISA showed calcitonin secretion from the differentiated C cell-like cells after 15 days differentiation (Figure 4f). We detected calcitonin secretion of 109 and 112 pg/mL (21.8 and 22.4 pg per calcitonin per 1x 10^5^ cells) from induced C cell-like cells in 3D, which represented a 2.4- and 2.2-fold increase in calcitonin secretion compared to that detected in cells from parallel 2D-induced cultures of H9 and HES3 hESCs, respectively (Figure 4f). Comparing the number of cells producing calcitonin in culture as measured by ELISA, 0.5–1 × 10^6^ cells in 2D against 1 × 10^5^ in 3D, we were able to infer that the number of cells producing calcitonin in 3D was approximately two times more than that in the 2D format. Thus, per every 10,000 cells, H9 hESC differentiated C cells released between 0.13–6.45 pg of calcitonin in the 2D format and 2.18–10.9 pg of calcitonin in 3D. This also further supports the idea that the cell or tissue differentiation is more efficient in a 3D format relative to 2D [75].

## 4. Discussion

Difference in the immune system, physiology, individual genetic backgrounds, and other factors makes the use of animals and animal cells as models for the study of the pathophysiology of many human diseases inadequate [22]. As a result of this, much attention has been directed recently to disease modelling using human pluripotent stem cells. Here, we have developed a novel protocol that allows for the generation of functional parafollicular cells (thyroid C cell-like cells) from hESCs. This complements a method for producing thyroid follicular-like cells from human pluripotent cells [26,64]. Combining the two induced lineages offers the potential to form a complete thyroid complement of cells.

Our results demonstrate the directed differentiation of hESC into DE cells and then AFE progenitors with thyroid C cell differentiation potential. The main objective of this protocol was to look for factors that will disfavour the differentiation of other cell lineages from the AFE and at the same time favour the differentiation of thyroid C cell-like cells expressing the required markers. The DE/thyroid C cell-like cell differentiation method was robust with little variability across three pluripotent cell lines (H9 and HES3 hESCs and 007 iPSCs) in the generation of calcitonin producing cells as assayed by PCR, immunolabelling, and ELISA.

To provide a temporally and spatially controlled environment which preferentially supports stem cell differentiation and maturation, researchers have used ECM-like collagen and Matrigel [72,76]. Here, we demonstrated the feasibility of differentiating thyroid C cells from hESCs using Matrigel as ECM. These 3D cultured cells produced and, on Ca^2+^ stimulation, released more calcitonin as analysed by ELISA than the cells in 2D culture, which was similar to previous findings that ECM supports stem cell differentiation and maturation [36,37,75,76].

In conclusion, these human thyroid C cell-like cells derived from pluripotent cells have the potential for investigating the pathogenesis and modelling of MTC and provide a platform for the screening of combinatorial drugs.

## Data Availability

The study did not report any data.

## Supplementary Materials

The following are available online at https://www.mdpi.com/article/10.3390/cells10112897/s1, Figure S1: (a) QPCR analysis of DE/AFE-like cells normalized to undifferentiated hiPSC (007 cell line), showing time 8 course upregulation of SOX17. No significant difference was recorded. N.D., not detected. *n* = 3 independent experiments. Error bars represent mean ± SEM. (b) QPCR analysis of DE/AFE-like cells normalized to undifferentiated hiPSC (007 cell line) and hESC (HES3), showing time course upregulation of NKX2.1. No significant difference was recorded. N.D., not detected. *n* = 3 independent experiments. Error bars represent mean ± SEM. (c) Immunofluorescence of differentiated thyroid C cell-like cells grown on laminin showing co-expression of C cell lineage markers, E-CADHERIN, CGRP, and PROCALCITONIN. Scale bar: 100 μm. (d) Immunofluorescence of differentiated thyroid C cell-like cells grown on Matrigel showing co-expression of C cell lineage markers, E-CADHERIN, CGRP, and PROCALCITONIN. Scale bar: 20 μm. (e) Immunofluorescence of differentiated thyroid C cell-like cells grown on Matrigel showing co-expression of C cell lineage markers, E-CADHERIN, ISL1, and PROCALCITONIN. Scale bar: 20 μm. Figure S2: (a) Wholemount staining of differentiated thyroid C cell-like cells in 3D Matrigel culture co-expressing E-CADHERIN, PROCALCITONIN, and ASCL1. Scale bar: 100 μm. (b) Wholemount staining of differentiated thyroid C cell-like cells in 3D matrigel co-expressing PGP9.5, E-CADHERIN, and CALCITONIN. Scale bar: 100 μm. (c) QPCR analysis of thyroid C cell-like cells normalized to differentiated DE/AFE (007 cell line), showing the downregulation of lung markers CC10 and SFTPC and upregulation of thyroid C cell-like cell, CALCA. No significant difference was recorded. N.D., not detected. *n* = 3 independent experiments. Error bars represent mean ± SEM. Figure S3: (a) Immunofluorescence of differentiated thyroid C cell-like cells grown on Laminin showing co-expression of C cell lineage markers NKX2.1 and PROCALCITONIN, and no expression of thyroid marker PAX8. Scale bar: 200 μm (upper) and 100 μm (lower). (b) Immunofluorescence of differentiated kidney cells as a positive control showing the specificity of the PAX8 antibody. Scale bar: 200 μm (upper) and 100 μm (lower). Table S1: Table of antibodies; Table S2: qPCR probes; Table S3: PCR primers for SYBR gene qPCR.
