# Supplementary material for: Development of Functional Thyroid C Cell-like Cells from Human Pluripotent Cells in 2D and in 3D Scaffolds
Source: Cells. 2021 Oct 26;10(11):2897. doi: 10.3390/cells10112897 (PMC8616516; doi:10.3390/cells10112897)
Supplement: Supplementary file 1 [file cells-10-02897-s001.zip › cells-1395994-supplementary.pdf]

cells

MDPI

# Supplementary Materials

**Table S1.** Table of Antibodies.

| Primary Antibodies | Target Type | Host/Type | Company | Dilution |
|---|---|---|---|---|
| Anti-Procalcitonin (ab166963) | C cells | Sheep polyclonal | Abcam | 1:200 |
| Anti-Calcitonin (A0576) | C cells | Rabbit polyclonal | Dako | 1:400–800 |
| Anti-FoxA2 [EPR4466] (ab108422) | DE/C cells | Rabbit monoclonal | Abcam | 1:300 |
| Anti-MASH1/Achaete-scute homolog 1 antibody (ab38556) | Neuronal | Rabbit polyclonal | Abcam | 1:500 |
| Anti-PGP9.5 (ab72910) | Neuronal | Chick Polyclonal | Abcam | 1:500 |
| Anti-Beta III Tubulin raised in chicken | Neuronal | Chick Polyclonal | MERCK PL | 1:1000 |
| Anti-E-cadherin (610181) | Epithelial | Mouse IgG2a | BD Biosciences | 1:200–1000 |
| Recombinant Anti-TTF1 antibody [EP1584Y] (ab76013) | Thyroid/C cells | Rabbit monoclonal | Abcam | |

| Secondary Antibodies | Host/Isotype | Company | Dilution |
|---|---|---|---|
| Anti-Sheep IgG:Alexa 488 (A-11015) | Donkey | ThermoFisher Sci. | 1:1000 |
| Anti-Sheep IgG:Alexa 594 (A-11016) | Donkey | ThermoFisher Sci. | 1:1000 |
| Anti-Rabbit IgG Alexa 488 (A-21206) | Donkey | ThermoFisher Sci. | 1:1000 |
| Anti-Rabbit IgG Alexa 594 (A-21207) | Donkey | ThermoFisher Sci. | 1:1000 |
| Anti-Rabbit IgG Alexa 633 (A-21070) | Goat | ThermoFisher Sci. | 1:1000 |
| Anti-Mouse IgG-specific:Alexa 488 (A-11001) | Goat | ThermoFisher Sci. | 1:1000 |
| Anti-Mouse IgG- specific:Alexa 488 (A-21202) | Donkey | ThermoFisher Sci. | 1:1000 |
| Anti-Mouse IgG+M Alexa 594 (A21203) | Donkey | ThermoFisher Sci. | 1:1000 |
| Anti-Mouse IgG Alexa 647 (A-31571) | Donkey | ThermoFisher Sci. | 1:1000 |
| Anti-Chick IgY Alexa 488 (703-545-155) | Donkey | Jackson Immunoresearch | 1:400–800 |
| Anti-Chick IgY Alexa 568 (A-11041) | Goat | ThermoFisher Sci. | 1:1000 |
| Anti-Chick IgY Alexa 488 (A-11039) | Goat | ThermoFisher Sci. | 1:1000 |

**Table S2.** qPCR probes.

| Probes | Target Type | Product Size | Company | Accession Number |
|---|---|---|---|---|
| *CDH1* (E-cadherin) | Cell-to-cell adhesion protein | 80 | ThermoFisher Sci | Hs01023895_m1 |
| *CDH2* (N-cadherin) | Cell-to-cell adhesion protein | 66 | ThermoFisher Sci | Hs00983056_m1 |
| *FOXA1* | Endodermal Marker | 59 | ThermoFisher Sci | Hs04187555_m1 |
| *FOXA2* | DE Marker | 66 | ThermoFisher Sci | Hs00232764_m1 |
| *RET* | Growth factor receptor | 83 | ThermoFisher Sci | Hs01120030_m1 |
| *GAPDH* | Housekeeper | 93 | ThermoFisher Sci. | Hs02758991_g1 |

**Table S3.** PCR primers for SYBR green qPCR.

| Gene | Target Type | Sequence 5′ to 3′ | Product Size |
|---|---|---|---|
| *GAPDH* | Housekeeper | F: GTCTCCTCTGACTTCAACAGCG<br>R: ACCACCCTGTTGCTGTAGCCAA | 131 bp |
| *TG* | Thyroid cell marker | F: CCAGTGGCTTCTCTTCCTGACT<br>R: CCTTGGAGGAAGCGGATGGTTT | 155 bp |
| *TSHR* | Thyroid Cell marker | F: GAGTTTCCTTCACCTCACACGG<br>R: CTGCTCTCATTACACATCAAGGAC | 114 bp |
| *RET* | Growth factor receptor | F: GAGGAGAGACTACTTGGACCTTG<br>R: GGGGACAGCGGTGCTAGAAT | 200 bp |
| *EPHA4* | Growth factor receptor | F: TTCTGCTATCTTGGCCTCACAG<br>R: TAGACGGAACTGAGGAGGGT | 85 bp |
| *CDX2* | Posterior endoderm/Intestinal differentiation | F: ACAGTCGCTACATCACCATCCG<br>R: CCTCTCCTTTGCTCTGCGGTTC | 107 bp |
| *NKX2.1* | DE Marker/Thyroid Transcription Factor | F: GCAACCTGGGCAACATGAG<br>R: CATGAAGCGGGAGATGGCG | 122 bp |
| *CER1* | DE marker | F: CAGGACAGTGCCCTTCAGCCA<br>R: ACAGTGAGAGCAGGAGGTATGG | 142 bp |
| *CALCA* | C-cell Marker | F: GTCATGGGCTTCCAAAAGTTC<br>R: CAGGGCAGACCTGAATGGTG | 99 bp |
| *PAX8* | Thyroid Follicular Marker | F: CGATGCCTCACAACTCCATCA<br>R: GCCAGGTCTACGATGCGCT | 115 bp |
| *FOXE1* | Thyroid differentiation marker | F: CTCAAACGGGATGCTTTCTGGT<br>R: GCACATGGAAGGCTGAAACTGA | 126 bp |

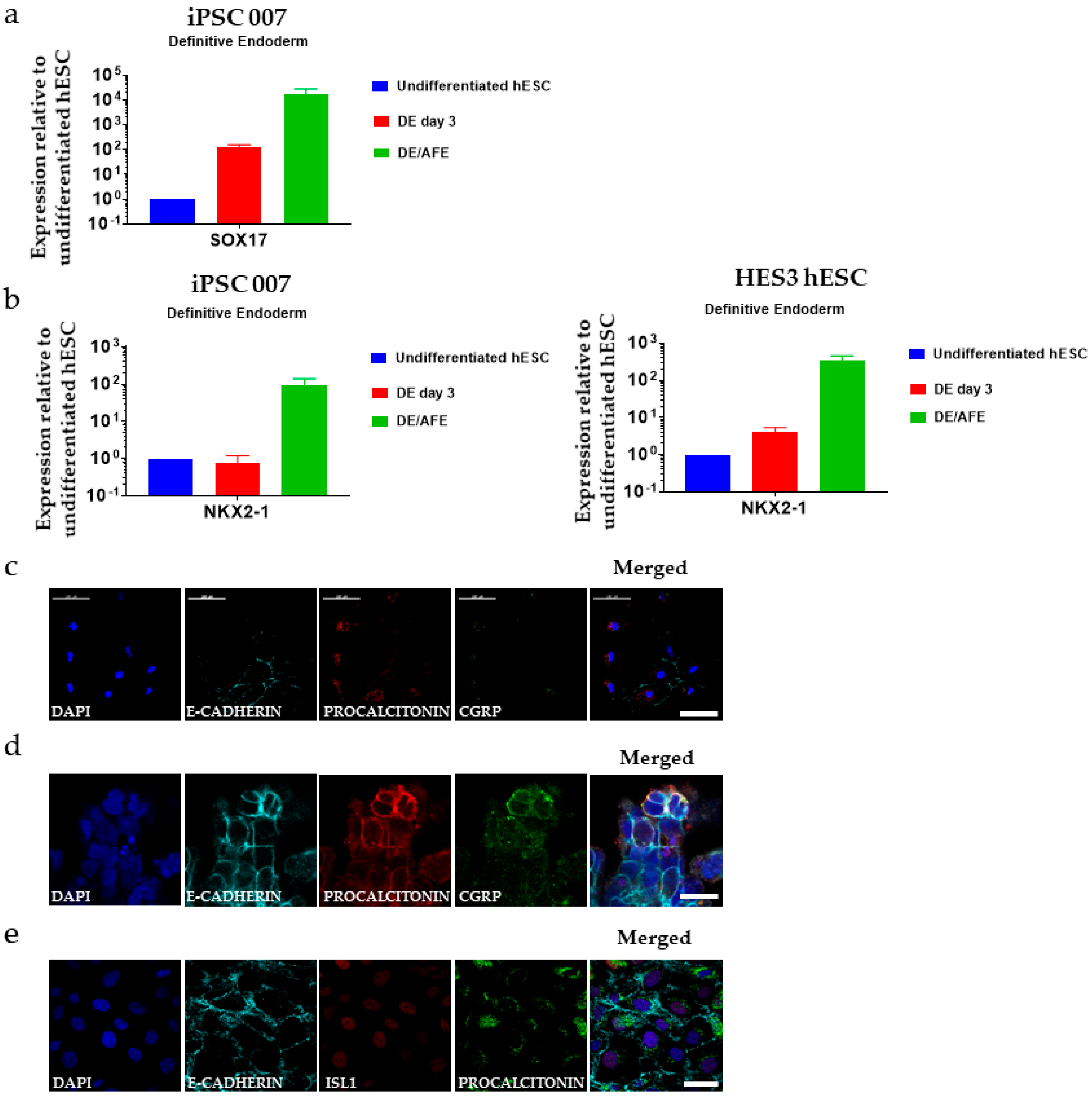


**Figure S1.** (**a**) QPCR analysis of DE/AFE-like cells normalized to undifferentiated hiPSC (007 Cell line), showing time course upregulation of *SOX17*. No significant difference was recorded. N.D.- Not Detected. *n* = 3 independent experiments. Error bars represent mean ± SEM. (**b**) QPCR analysis of DE/AFE-like cells normalized to undifferentiated hiPSC (007 Cell line) and hESC (HES3), showing time course upregulation of *NKX2.1*. No significant difference was recorded. N.D.- Not Detected. *n* = 3 independent experiments. Error bars represent mean ± SEM. (**c**) Immunofluorescence of differentiated thyroid C cell-like cells grown on laminin showing co-expression of C cell lineage markers, E-CADHERIN, CGRP and PROCALCITONIN. Scale bar: 100 µm. (**d**) Immunofluorescence of differentiated thyroid C cell-like cells grown on Matrigel showing co-expression of C cell lineage markers, E-CADHERIN, CGRP and PROCALCITONIN. Scale bar: 20 µm. (**e**) Immunofluorescence of differentiated thyroid C cell-like cells grown on Matrigel showing co-expression of C cell lineage markers, E-CADHERIN, ISL1 and PROCALCITONIN. Scale bar: 20 µm.

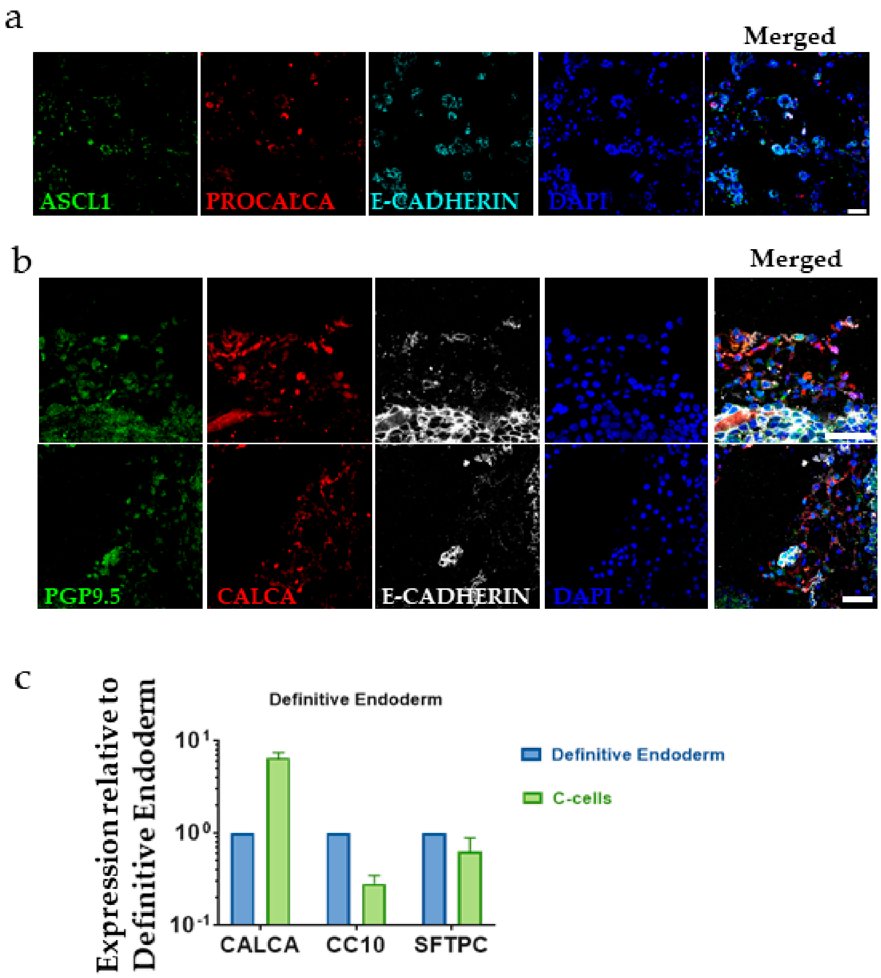


**Figure S2.** (**a**) Wholemount staining of differentiated thyroid C cell-like cells in 3D Matrigel culTable 1. Scale bar: 100 µm. (**b**) Wholemount staining of differentiated thyroid C cell-like cells in 3D Matrigel co-expressing PGP9.5, E-CADHERIN and CALCITONIN. Scale bar: 100 µm. (**c**) QPCR analysis of thyroid C cell-like cells normalized to differentiated DE/AFE (007 Cell line), showing the downregulation of lung markers- *CC10* and *SFTPC* and upregulation of thyroid C cell-like cells-*CALCA.* No significant difference was recorded. N.D.- Not Detected. $n = 3$ independent experiments. Error bars represent mean ± SEM.

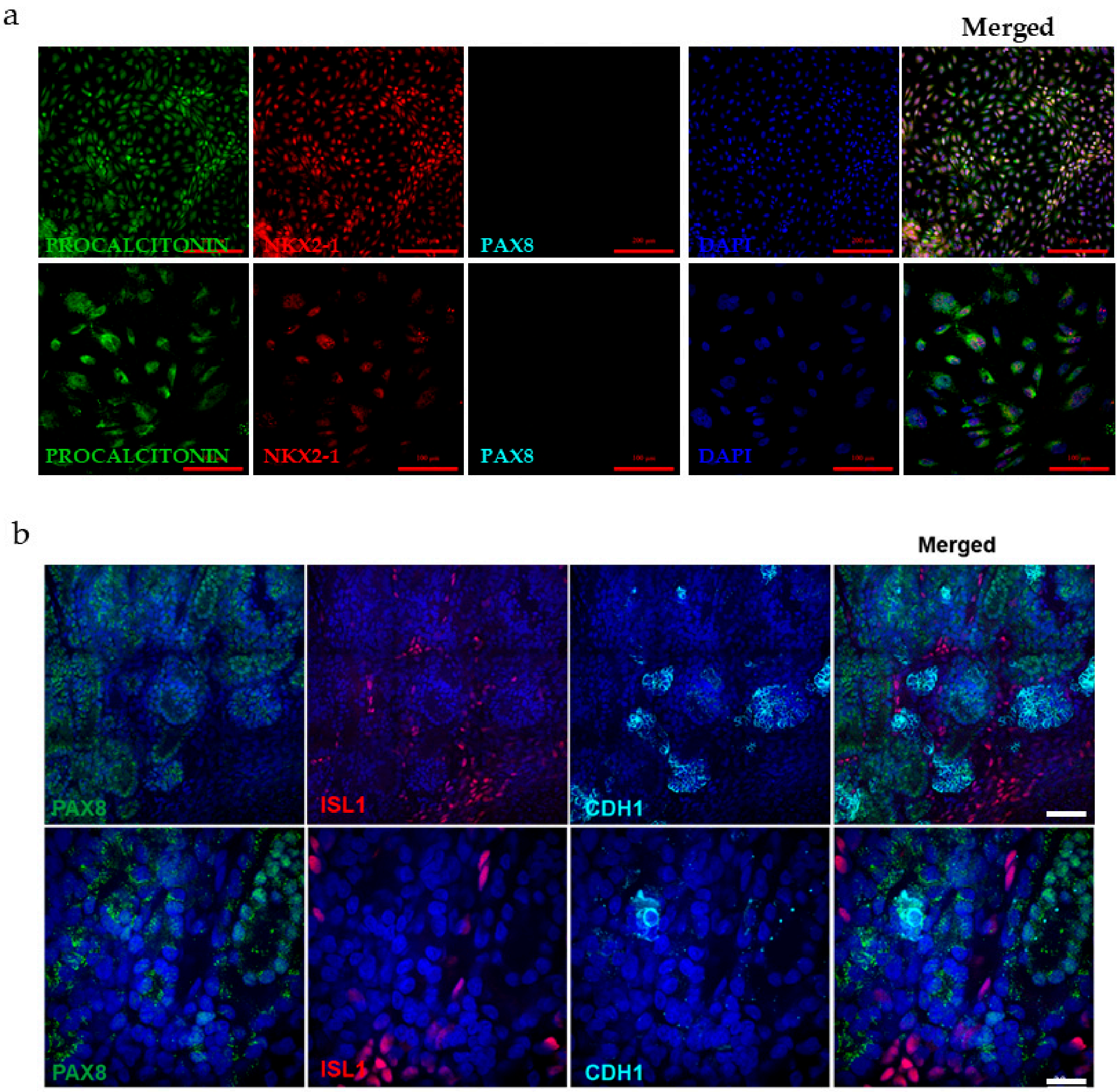


**Figure S3.** (**a**) Immunofluorescence of differentiated thyroid C cell-like cells grown on Laminin showing co-expression of C cell lineage markers, *NKX2.1 and PROCALCITONIN* and no expression of thyroid marker, *PAX8*. Scale bar: 200 µm (upper) and 100 µm (lower). (**b**) Immunofluorescence of differentiated kidney cells as a positive control showing the specificity of the *PAX8* antibody. Scale bar: 200 µm (upper) and 100 µm (lower).
